# Effects of preoperative inflammation parameters on regional anesthesia blockade times in pregnant women

**DOI:** 10.12669/pjms.41.9.12144

**Published:** 2025-09

**Authors:** Dilek Yeniay, Ali Altınbas, Mehmet Degermenci, Hilmi Furkan Arslan

**Affiliations:** 1 Dilek Yeniay Department of Anesthesiology and Reanimation, Giresun Training and Research Hospital, Giresun, Turkey; 2 Ali Altınbas Department of Anesthesiology and Reanimation, Giresun Training and Research Hospital, Giresun, Turkey; 3 Mehmet Degermenci Department of Anesthesiology and Reanimation, Giresun Maternity and Child Health Training and Research Hospital, Giresun, Turkey; 4 Hilmi Furkan Arslan Department of Medical Biochemistry, Giresun Maternity and Child Health Training and Research Hospital, Giresun, Turkey

**Keywords:** Monocyte-to-lymphocyte ratio, Monocyte-to-platelet ratio, Neutrophil-to-lymphocyte ratio, Pan-immune inflammation value, Platelet-to-lymphocyte ratio, Systemic immune inflammation index, Systemic inflammation response index

## Abstract

**Objective:**

We aimed to investigate the effects of inflammatory markers measured in pregnant women before cesarean section on the duration of sensory and motor block of local anesthetics used in regional anesthesia.

**Methodology:**

This prospective study was conducted at Giresun Obstetrics and Pediatrics Education and Research Hospital on patients who underwent cesarean section surgery between March 15, 2023, and July 15, 2023. Neutrophil, lymphocyte, platelet and monocyte counts were recorded in the preoperative hemograms of the patients. Neutrophil to lymphocyte ratio (NLR), monocyte to lymphocyte ratio (MLR), platelet to lymphocyte ratio (PLR), monocyte to platelet ratio (MPV), systemic immune inflammation index (SII), systemic inflammation response index (SIRI) and pan-immune inflammation value (PIV) were calculated. The duration of sensory block, Modified Bromage scale duration for motor block (MB1-2-3) and the duration of sensory (SBD) and motor (MBD) block reversal in the postoperative period were recorded in patients who underwent spinal anesthesia.

**Results:**

A total of 79 patients were included in the study. Statistically significant differences were found between NLR and MB2, MB3 and SBD durations, between SII and MB1 and MB2 durations, between SIRI and MB3 duration, between PIV and MB1 duration, between PLR and MB1 and MB2 durations, between PLR and MB1 and motor block durations, between MPV and MB3 (p<0.05).

**Conclusion:**

The use of simple and accessible biomarkers can be a valuable guide in anesthesia planning. Especially in patients with high levels of inflammation, the dose, type or method of administration of local anesthetics may be revised.

## INTRODUCTION

Cesarean delivery is the most common obstetric operation performed worldwide. The cesarean section rate, which was around 5% in 1965 in developed countries, increased five fold in the 1990s and has been reduced to 20% with various measures.^1^ The anesthesia method chosen in cesarean section interventions has a special place in anesthesia because of its direct effect on the newborn. The anesthesiologist should choose the anesthesia method that is the safest and most comfortable for the mother, has the least side effects on the newborn and provides optimal working conditions for the surgery. Recently, regional anesthesia methods have been increasingly preferred in cesarean sections because they have many advantages such as the fact that newborns are not exposed to the effects of anesthetic drugs, the risk of aspiration is low, and the mother is awake during and after surgery, thus allowing early establishment of the emotional bond between mother and baby.^2^

Many factors including gender, weight, height, intra-abdominal pressure and spinal anatomy have been defined to affect the intrathecal spread of local anesthetics used in regional anesthesia.^3^ In addition, it is known that the presence of inflammation decreases the effect of local anesthetics as a result of changes in local tissue pH, blood flow, nociceptors and central nervous system.^4^ However, there is not enough data in the literature about the effect of inflammation in the body on local anesthetics.

Neutrophil-to-lymphocyte ratio (NLR), monocyte-to-lymphocyte ratio (MLR), platelet-to-lymphocyte ratio (PLR), and monocyte-to-platelet ratio (MPV) are inflammatory biomarkers frequently used as prognostic factors in various diseases.^5^-^8^ Recently, new and comprehensive inflammation markers derived from blood cell counts such as systemic immune inflammation index (SII), systemic inflammation response index (SIRI) and pan-immune inflammation value (PIV) have also attracted attention.^8^

In this study, we aimed to investigate the effects of inflammatory markers on the duration of sensory and motor block of local anesthetics used in regional anesthesia before cesarean section in pregnant women.

## METHODOLOGY

This study was conducted prospectively at Giresun Obstetrics and Pediatrics Training and Research Hospital, between March 15, 2023 and July 15, 2023’

A pilot study was performed on 30 patients using the ’correlation: bivariate normal model’ test with G power software. In our study, the r-value was 0.346. In the light of these data, sample size analysis was performed using a=0.05, 80% power. The sample size was calculated as 63 patients in total. Considering the dropout rate, a total of 79 patients were included in the study.

### Ethical Approval:

It was obtained from the Ethics Committee (Decision No-50/2023; dated: March 13, 2023) and institutional study permissions were obtained.

The procedures to be performed were explained to the pregnant women who agreed to participate in the study and their verbal and written consents were obtained. Pregnant women with a body mass index of 25-35 kg/m2, aged 18-40 years, who were assigned an ASA II risk index solely due to pregnancy and underwent elective cesarean section were included in our study. Patients with ASA III and above, pregnant women with a contraindication to spinal anesthesia such as infection or coagulation disorder at the needle insertion site and pregnant women undergoing surgery under emergency conditions were excluded.

### Anesthesia management:

Standard monitoring (noninvasive blood pressure, electrocardiogram, peripheral oxygen saturation) was performed. After 12 mg bupivacaine (Bupivon, spinal heavy 0.5%, Onfarma, Turkey) was administered into the spinal space with a 25 Gauge Quincke-tipped needle through the L4-5 vertebral space of the patient who was placed in a sitting position, the patient was quickly moved to a 15 degree left lateral recumbency position. The level of sensory block was checked with a pinprick test and the level of motor block was evaluated with the Modified Bromage (MB) scale (0: No paralysis, the patient can fully flex the foot and knee, 1: The patient can only move the knee and foot, but cannot lift the leg straight, 2: The patient cannot flex the knee and can only move the foot, 3: The patient cannot move the foot joint or the big toe, there is complete paralysis). Surgery was initiated when the sensory block reached T6 dermatome level. If there was no evidence of anesthesia within the first 10 minutes after intrathecal injection, it was considered a technical failure, and these patients underwent general anesthesia and were excluded from the study. Mean arterial pressure (MAP), heart rate (HR) and peripheral oxygen saturation (SpO2) were recorded before the procedure (baseline), at three minutes, five minutes, 10 minutes, 15 minutes, 30 minutes and 45 minutes after the procedure. Time to sensory block at T6 dermatome, time to reach each score of the MB scale (MB1-MB2-MB3), total operative time (from the beginning of the surgical incision to the completion of the surgery), the highest level of sensory block, and anesthetic complications such as hypotension and bradycardia were recorded. Patients were taken to the recovery unit at the end of surgery and transferred to the surgical ward when the sensory block level was below the T6 dermatome and the Modified Aldrete score was above 8. The duration of sensory block (SBD) (time from local anesthetic injection to S2 dermatome sensation) and motor block (MBD) (time from local anesthetic injection to full motor function recovery) were recorded.

### Measures and Grouping of the patients:

Neutrophil, lymphocyte, platelet and monocyte counts in the hemograms of the patients, which were routinely studied before surgery, were recorded. According to these inflammation parameters, NLR = neutrophil/lymphocyte ratio, SII = plateletxneutrophil/lymphocyte ratio, SIRI = neutrophilxmonocyte/lymphocyte ratio, PIV = neutrophilxtrombocytexmonocyte/lymphocyte ratio, PLR = platelet/lymphocyte ratio, MLR = monocyte/lymphocyte ratio and MPV = monocyte/platelet ratio were calculated. For these ratios, cut off values determined using ROC analysis in previous studies in the literature were determined and patients were divided into groups according to these values. The accepted cut off values were four for NLR,^5^,^7^ 750 for SII,^9^ 2.55 for SIRI,^10^ 428 for PIV,^11^ 121 for PLR,^6^ 0.25 for MLR^12^ and 9.55 for MPV.^13^

### Statistical Analysis:

All statistical analyses were performed using SPSS version 26.0 (IBM Corp, Armonk, New York, United States). The Kolmogorov–Smirnov test was used to assess data normality. Continuous data with a normal distribution (parametric) are reported as Mean (Standard Deviation), while non-normally distributed data (non-Parametric) are reported as Median (25th–75th percentile). In comparing the clinical data and biochemical data of the patients, the Mann-Whitney U test was used for nonparametric variables and the Student t-test was used for parametric variables. Pearson correlation analysis was applied for the parameters that were significant between the groups.

## RESULTS

Patients were divided into two groups as low and high according to the cut off values accepted in the literature for NLR, SII, SIRI, PIV, PLR, MLR, MPV parameters. No statistically significant difference was found between the groups formed according to all parameters in terms of age, weight and height (p>0.05 for all) (Table-I). Basal and 3rd minute heart rates were found to be statistically significantly higher in the NLR>4 group compared to the low group (p = 0.018 and p = 0.006, respectively). While MB2 and MB3 measurements of NLR>4 patients were statistically lower compared to NLR<4 patients (p = 0.029 and p = 0.039, respectively), no significant difference was found between MB1 measurements (p = 0.477). Although the time to reach the T6 level was numerically higher in the NLR>4 group, no statistically significant difference was found (p=0.657). In the postoperative period, SBD measurement was significantly higher in the NLR>4 group (p = 0.031), whereas no significant difference was found between the groups in MBD measurement (p = 0.317) (Table-I).

**Table-I:** Comparison of laboratory data and clinical findings (NLR, PLR, MLR, MPV).

	NLR<4 (N=37)	NLR >4 (N=42)	p-value	PLR<121 (N=46)	PLR>121 (N=33)	p-value	MLR<0.25 (N=25)	MLR>0.25 (N=54)	p-value	MPV<9.55 (N=40)	MPV>9.55 (N=39)	p-value
Age	31.18±5.67	30.92±3.94	0.812	31.39±5.31	30.57±4.01	0.409	30,76±5,87	31.18±4,26	0,717	31,02±5.21	31.10±4.40	0.925
Weight	80.75±10.76	80.66±9.95	0.969	80.96±10.17	80.36±10.55	0.802	82.76±9.86	79.75±10.46	0.230	79.90±10.41	81.53±10.18	0.482
Boy	161.35±5.16	162.07±4.81	0.523	161.4±5.02	162.1±4.90	0.558	160.8±4.80	161.9±5.06	0.898	161.3±5.18	162.17±4.72	0.434
Basal HR	96.12±12.96	105.28±17.07	0.018*	93.67±15.41	101.78±17.23	0.011*	98.12±10.83	107.3±11.89	0.047*	99.17±16.04	102.6±16.32	0.161
HR 3.min	94.97±17.16	108.45±25.21	0.006*	99.84±18.22	103.1±20.16	0.182	100.2±19.37	105.1±20.34	0.142	95.51±21.18	103.3±24.66	0.044*
HR 5.min	97.04±25.03	103.18±24.30	0.058	97.04±22.1	99.84±20.38	0.307	99.24±24.02	101.9±25.01	0.417	84.61±16.38	98.11±15.13	0.025*
HR 10.min	86.67±15.06	89.38±17.99	0.474	93.72±14.56	95.33±18.66	0.326	94.20±11.73	96.04±13.68	0.528	91.01±13.87	86.24±12.55	0.523
HR 15.min	91.43±11.41	92.42±14.47	0.734	91.65±11.82	92.39±14.75	0.805	92.72±11.93	90.11±11.64	0.691	89.02±12.88	92.84±12.23	0.556
HR 30.min	90.33±13.94	89.83±12.74	0.877	91.73±13.78	87.48±12.11	0.728	89.57±10.04	89.71±11.15	0.833	92.08±13.45	91.77±14.85	0.813
HR 45.min	82.73±14.54	87.19±10.64	0.512	85.16±12.41	84.83±10.61	0.611	89.38±13.67	86.55±12.08	0.391	84.14±18.67	85.76±12.11	0.884
MB1 (sec)	109.8±37.93	103.1±36.71	0.277	111.2±32.17	91.93±21.85	0.038*	119.4±31.36	99.13±36.14	0.038*	115.2±36.08	107.1±38.13	0.431
MB2 (sec)	192.2±30.71	148.8±30.75	0.029*	180.2±61.85	146.5±62.73	0.026*	192.1±61.56	166.3±60.33	0.119	178.7±66.19	170.4±70.24	0.640
MB3 (sec)	298.5±63.46	235.5±47.92	0.039*	298.7±66.14	262.2±55.61	0.107	293.3±81.25	267.3±87.3	0.257	290.2±70.13	230.1±80.36	0.048*
Duration of surgery (min)	43.21±17.11	41.76±16.44	0.805	44.26±16.67	41.18±16.73	0.421	46.56±18.24	41.31±15.76	0.195	42.01±16.26	43.97±17.20	0.602
SBD (min)	115(90-155)	147(96-190)	0.031*	123(93-182)	115(88-177)	0.231	122(105-200)	117(85-178)	0.296	120(90-177)	125(92-185)	0.731
MBD (min)	160(90-190)	152(106-210)	0.317	167(101-202)	135(105-187)	0.496	185(123-210)	135(93-191)	0.045*	157(87-206)	150(106-190)	0.579
T6 transportation time (sec)	180(120-240)	165(135-235)	0.657	180(120-240)	160(120-240)	0.504	180(120-255)	180(127-236)	0.845	180(120-255)	180(120-240)	0.783
Highest sensory block level	T4 (4-5)	T4 (4-5)	0.702	T4 (4-5)	T4 (4-5)	0.711	T4 (4-5)	T4 (4-5)	0.790	T4 (4-5)	T4 (4-5)	0.699

NLR: Neutrophil to lymphocyte ratio. PLR: platelet to lymphocyte ratio. MLR: monocyte to lymphocyte ratio. MPV: monocyte to platelet ratio. HR: heart rate. MB: modifiye bromage. SBD: sensory block duration. MBD: motor block duration.

* p<0.05 statistical significance. p values were obtained by Mann-Whitney U and Student's t tests. Parametric data are presented as Mean±SD. nonparametric data as Median (25th-75th percentile).

In the PLR>121 group, only basal heart rate was found to be statistically significantly higher than in the low group (p=0.011), whereas no significant difference was found in heart rates in the other minutes (p>0.05 for all). MB1 and MB2 measurements of PLR>121 patients were statistically significantly lower compared to PLR<121 patients (p=0.038 and p=0.026, respectively), whereas no significant difference was found in MB3 measurements (p=0.107) (Table-I).

In the group with MLR>0.25, only basal heart rate was found to be statistically significantly higher than the group with lower MLR (p=0.047), whereas no significant difference was found in heart rates in the other minutes (p>0.05 for all). When the MB1 measurements of MLR>0.25 patients were compared with MLR<0.25 patients, statistically significantly lower results were found (p=0.038), while no significant difference was found between the groups in MB2 and MB3 measurements (p=0.119 and p=0.257, respectively) (Table-I).

In the group with MPV>9.55, heart rates at three and five minutes were statistically significantly higher than in the group with lower MPV (p = 0.044 and p = 0.025, respectively). MB3 measurements of MPV>9.55 patients were statistically significantly lower than those of MPV<9.55 patients (p = 0.048), whereas no significant difference was found between the groups in MB1 and MB2 measurements (p = 0.431 and p = 0.640, respectively) (Table-I).

In the SII>750 group, heart rate was statistically significantly higher only in the 3rd minute compared to the low SII group (p=0.026), whereas there was no significant difference in heart rates in the other minutes (p>0.05 for all) (Table-I). MB1 and MB2 measurements of SII>750 patients were statistically significantly lower compared to SII<750 patients (p=0.020 and p=0.035, respectively), whereas no significant difference was found between MB3 measurements (p=0.316) (Table-II).

**Table-II:** Comparison of laboratory data and clinical findings (SII. SIRI. PIV).

	SII<750 (N=29)	SII>750 (N=50)	p-value	SIRI<2.55 (N=53)	SIRI>2.55 (N=26)	p-value	PIV<428 (N=33)	PIV>428 (N=46)	p-value
Age	31.68±5.71	30.68±4.21	0.410	30.81±5.24	31.53±3.79	0.530	31.54±5.66	30.69±4.09	0.441
Weight	79.62±11.01	81.34±9.87	0.491	80.43±9.69	81.26±10.54	0.737	80.42±9.58	80.91±9.15	0.836
Boy	161.17±5.52	162.06±4.62	0.446	161.4±5.03	162.2±4.85	0.536	160.6±4.94	162.5±10.15	0.196
Basal HR	98.04±14.02	103.98±17.27	0.074	100.9±15.13	102.8±18.20	0.198	100.7±14.52	103.4±17.16	0.132
HR 3.min	95.82±19.41	105.88±25.60	0.026*	99.03±24.86	105.9±22.82	0.042*	102.5±19.69	105.1±20.61	0.190
HR 5.min	97.58±25.32	100.54±24.39	0.160	93.19±25.37	102.52±23.46	0.023*	91.15±19.72	102.5±20.14	0.007*
HR 10.min	86.58±15.06	90.16±17.29	0.152	87.03±16.42	90.31±17.17	0.415	89.43±20.21	91.24±19.36	0.113
HR 15.min	91.00±9.77	92.52±14.69	0.583	90.92±12.85	92.47±13.65	0.623	91.48±20.65	92.87±21.77	0.547
HR 30.min	90.96±11.64	89.53±14.21	0.652	91.06±12.78	87.95±14.36	0.367	90.23±18.91	89.68±15.44	0.613
HR 45.min	86.9±13.67	84.02±20.96	0.683	85.39±12.13	84.01±17.34	0.758	88.13±13.46	84.91±12.07	0.397
MB1 (sec)	120.6±39.85	97.84±34.91	0.020*	108.7±33.85	99.76±28.13	0.125	119.4±39.65	98.93±35.26	0.019*
MB2 (sec)	189.7±55.51	155.6±43.72	0.035*	178.9±51.24	165.3±59.72	0.108	182.5±47.96	158.7±35.41	0.076
MB3 (sec)	289.4±93.86	267.7±75.37	0.316	278.5±88.32	236.3±74.85	0.044*	280.6±82.21	272.2±83.31	0.252
Duration of surgery (min)	42.41±17.97	43.30±16.02	0.827	44.01±17.24	40.84±13.87	0.389	44.30±17.51	42.02±16.14	0.557
SBD (min)	120(95-175)	123(90-182)	0.939	117(81-171)	122(95-187)	0.398	120(97-183)	121(90-176)	0.680
MBD (min)	180(88-209)	149(105-191)	0.180	160(99-209)	135(89-184)	0.475	165(98-209)	149(93-195)	0.547
T6 transportation time (sec)	180(120-230)	180(120-240)	0.740	180(120-240)	165(127-236)	0.675	180(120-235)	180(127-245)	0.664
Highest sensory block level	T4 (4-5)	T4 (4-5)	0.749	T4 (4-5)	T4 (4-5)	0.668	T4 (4-5)	T4 (4-5)	0.696

SII: systemic immune inflammation index. SIRI: systemic inflammation response index. PIV: pan-immune inflammation value. HR: heart rate. MB: modifiye bromage. SBD: sensory block duration. MBD: motor block duration

* p<0.05 statistical significance. p values were obtained by Mann-Whitney U and Student’s t tests. Parametric data are presented as Mean±SD. nonparametric data as Median (25th-75th percentile).

Heart rates at three and five minutes were found to be statistically significantly higher in the SIRI>2.55 group compared to the low SIRI group (p = 0.042 and p = 0.023, respectively). When the MB3 measurements of SIRI>2.55 patients were compared with SIRI<2.55 patients, statistically significantly lower results were found (p = 0.044), while no significant difference was found between the groups in MB1 and MB2 measurements (p = 0.125 and p = 0.108, respectively) (Table-II).

In the PIV>428 group, heart rate was found to be statistically significantly higher only in the 5th minute compared to the low PIV group (p=0.007), whereas there was no significant difference in heart rates in the other minutes (p>0.05 for all). MB1 measurements of PIV>428 patients were statistically significantly lower compared to PIV<428 patients (p=0.019), whereas no significant difference was found between the groups in MB2 and MB3 measurements (p=0.076 and p=0.252, respectively) (Table-II).

When the groups formed according to all parameters were evaluated in terms of OAB, SpO2, operation times, T6 access times and the highest sensory block level, no statistically significant difference was found between the groups (p>0.05 for all).

The statistically significant results of Pearson correlation analysis between preoperative NLR and baseline HR, 3rd minutes HR, MB2s, MB3s, SBD parameters are presented in Table-III. There were weak/moderate positive correlations between preoperative NLR and 3rd minutes HR and postoperative NLR (r = 0.339 and p = 0.013, r = 0.586 and p = 0.007, respectively), while a weak negative correlation was observed between MB2 (r = - 0.245 and p = 0.041).

**Table-III:** Significant parameters in Pearson correlation analysis between systemic inflammation markers and clinical parameters.

Parameters 1	Parameters 2	Correlation coefficients	
NLR	HR 3.min	r = 0.339	p = 0.013
	MB2	r = - 0.245	p = 0.041
SII	HR 3.min	r = 0.211	p = 0.046
	MB1	r = - 0.294	p = 0.028
SIRI	HR 5.min	r = 0.266	p = 0.039
PIV	HR 5.min	r = 0.463	p = 0.015
	MB1	r = - 0.281	p = 0.027
PLR	HR basal	r = 0.315	p = 0.021
	MB2	r = - 0.257	p = 0.036
MLR	MB1	r = - 0.226	p = 0.043
MPV	HR 5.min	r = 0.275	p = 0.032

NLR: Neutrophil to lymphocyte ratio. MLR: monocyte to lymphocyte ratio. PLR: platelet to lymphocyte ratio. MPV: monocyte to platelet ratio. SII: systemic immune inflammation index. SIRI: systemic inflammation response index. PIV: pan-immune inflammation value. HR: heart rate. MB: modifiye bromage. *p<0.05 statistical significance, r: Pearson Correlation coefficient.

The significant results of the correlation analysis between SII and 3rd minutes HR, MB1, MB2 parameters are shown in Table-III. There was a weak positive correlation between SII and 3rd minutes HR (r = 0.211 and p = 0.046), while there was a weak negative correlation between MB1 (r = - 0.294 and p = 0.028). The significant results of the correlation analysis between SIRI and three- and five-minutes HR and MB3 parameters are presented in Table-III. SIRI was weakly positively correlated with 5-minutes HR (r = 0.266 and p = 0.039). The significant results of the correlation analysis between PIV and 5-minutes HR and MB1 parameters can also be seen in Table-III. There was a positive, weak correlation between PIV and 5-minutes HR (r = 0.463 and p = 0.015), and a weak negative correlation between MB1 (r = - 0.281 and p = 0.027).

The significant results of the correlation analysis between PLR and basal HR, MB1 and MB2 parameters are presented in Table-III. PLR was positively and weakly correlated with basal HR (r = 0.315 and p = 0.021), while MB2 was weakly negatively correlated (r = - 0.257 and p = 0.036). The significant results of the correlation analysis between MLR and basal HR, MB1 and MBD parameters are also presented in Table-III. A weak negative correlation was found between MLR and MB1 (r = -0.226 and p = 0.043). The significant results according to the correlation analysis between MPV and 3rd and 5th minute HR and MB3 parameters are also highlighted in Table-III. There was a weak positive correlation between MPV and 5-minutes HR (r = 0.275 and p = 0.032).

## DISCUSSION

In this study, we investigated the efficacy of inflammation markers on maternal hemodynamics and regional anesthesia blockage times in pregnant patients undergoing cesarean section. Since neutrophil and platelet levels increase in response to acute inflammation and lymphocyte levels decrease due to physiologic stress, inflammation markers can be used in the prognosis of some inflammatory diseases.^14^ Neutrophil, lymphocyte and platelet values measured by simple blood tests alone have low reliability in recognizing systemic inflammation. Therefore, indices combining more than one inflammation parameter have been developed. The efficacy of inflammation markers such as NLR, MLR, PLR and MPV has been proven by many studies and in addition to these, new inflammation markers such as SII, SIRI and PIV have recently been used.^8^,^15^

NLR and PLR values, which are evaluated by cardiologists for the risk of mortality after major cardiac events, have also been evaluated as markers with prognostic significance for diseases such as acute coronary syndrome, diabetes, Sjögren’s disease, ulcerative colitis and Guillain-Bare syndrome.^14^ Yildiz et al.^16^ showed that PLR was an effective parameter in predicting reperfusion failure in ST-segment elevation myocardial infarction patients. In a study by Altınbaş et al.^13^ examining the relationship between hemogram parameters and postoperative complications, it was stated that preoperative PLR could be used as a guide to predict the development of bronchospasm and laryngospasm and MPV values could be used as a guide to predict the risk of hypotension.

Karabay et al.^8^ reported that inflammatory markers including NLR, PLR, MLR, SII, SIRI, PIV, IG and MII are reliable potential markers in predicting delivery within 72 hours in the latent period in pregnancies with preterm premature rupture of membranes. The inflammatory process, which increases during pregnancy, may deepen with cesarean section and anesthesia management.^17^,^18^ Therefore, evaluation of the effect of inflammation markers on anesthesia outcomes may provide important contributions to clinical practice.

In addition to patient characteristics such as age, height, weight and gender, many factors such as intra-abdominal pressure, spinal anatomy, pregnancy, patient position, needle type, injection level and especially the baricity, density and dose of local anesthetic solutions may affect the spread of local anesthetics in the cerebrospinal fluid.^19^ In addition, it is thought that hormonal and immunologic changes that occur during pregnancy may have specific effects on the efficacy of local anesthetics.^20^ Studies examining the relationship between local anesthetics and inflammation show that inflammation decreases tissue pH and as a result, the ionized form of local anesthetics increases. It is thought that the efficacy of local anesthetics in the target tissue may decrease by this mechanism.^4^

However, there are no studies in the literature evaluating the relationship between systemic inflammation values and the effect of local anesthetics as in our study. The results obtained in our study showed that inflammation levels may play an important role in regional anesthesia management and local anesthetic efficacy. It was observed that modified bromage times were shorter and sensory block times were prolonged in groups with high inflammation levels. This indicates the effect of systemic inflammation on the pharmacodynamics of local anesthetics and suggests that inflammation may have a different effect on local anesthetics contrary to what is reported in the literature. In particular, systemic inflammation may have increased the efficacy of local anesthetics through mechanisms that alter the biochemical activity of local anesthetics in nerve cells, rather than through effects on local tissue pH or blood flow.

In our study, although there was no significant difference between the groups in terms of mean arterial pressure, heart rate was generally higher in the groups with higher levels of inflammation. The statistically significant higher heart rates at three and five minutes after spinal anesthesia support the relationship between cardiovascular complications and inflammatory markers shown in many studies. This is important for us to be more careful in pregnant patients with physiologically high heart rates.

### Limitations:

This study has the limited generalizability of the results due to the evaluation of this study in a specific population (pregnant women undergoing elective cesarean section). Furthermore, no molecular level analysis was performed to explain the mechanisms of the effect of inflammation on local anesthetics.

Studies with large populations and covering different clinical situations are needed to examine the relationship between local anesthetics and inflammation parameters in more detail. In our study, we used bupivacaine, which is the most commonly used local anesthetic in pregnant women. Randomized controlled studies evaluating the interactions of different types of local anesthetics with inflammation and their pharmacokinetic differences may fill the knowledge gaps in this field.

## CONCLUSION

The use of simple and accessible biomarkers, as in this study, can be a valuable guide in anesthesia planning. Understanding the effects of inflammation parameters on local anesthetics enables more effective assessment of patients in terms of both intraoperative hemodynamics and postoperative pain management and mobilization in regional anesthesia applications. In particular, in patients with high inflammation levels in the preoperative period, the dose, type, or method of administration of local anesthetics may be reconsidered.

### Author’s Contributions:

**DY, AA:** Literature search, Concept, design, Critical Review. **DY, MD:** Data Collection and r Processing. Critical review. **AA, HFA:** Critical Analysis, Interpretation. Critical review. All authors have read and approved the final fersion. Theya re also jointly responsible for ensuring integrity, and reliability of the study.
